# Dysregulation of COVID-19 related gene expression in the COPD lung

**DOI:** 10.1186/s12931-021-01755-3

**Published:** 2021-05-29

**Authors:** Alastair Watson, Lisa Öberg, Bastian Angermann, C. Mirella Spalluto, Michael Hühn, Hannah Burke, Doriana Cellura, Anna Freeman, Daniel Muthas, Damla Etal, Graham Belfield, Fredrik Karlsson, Karl Nordström, Kris Ostridge, Karl J. Staples, Tom Wilkinson, the MICA II Studygroup, Bastian Angerman, Stephanie Ashenden, Sarah Bawden, Graham Belfield, Maria G. Belvisi, Aurelie Bornot, Jerome Bouquet, Hannah Burke, Carolina Caceres, Raghothama Chaerkady, Doriana Cellura, Chia-Chien Chiang, Kerry Day, Antonio DiGiandomenico, Hanna Duàn, Ulrika Edvardsson, Damla Etal, Anna Freeman, Matthew Glover, Vancheswaran Gopalakrishnan, Stephen Harden, Sonja Hess, Alex Hicks, Ventzi Hristova, Michael Hühn, Fredrik Karlsson, Shameer Khader, Glenda Lassi, Alex Mackay, Chris McCrae, Christopher Morehouse, Daniel Muthas, Karl Nordström, Steven Novick, Esther Nyimbili, Kristoffer Ostridge, Lisa Öberg, Adam Platt, Laura Presland, Xiaotao Qu, Nicola Rayner, Pedro Rodrigues, Bret Sellman, Gary Sims, Cosma Mirella Spalluto, Andria Staniford, Karl J. Staples, Bruce Thompson, Junmin Wang, Paul Warrener, Alastair Watson, Nicholas P. Williams, Tom M. A. Wilkinson, Wen Yu, Bairu Zhang, Tianhui Zhang, Natalie van Zuydam

**Affiliations:** 1 Faculty of Medicine, University of Southampton, Southampton, UK; 2 NIHR Southampton Biomedical Research Centre, University Hospital Southampton, Southampton, UK; 3 Translational Science and Experimental Medicine, Research and Early Development, AstraZeneca, Respiratory & Immunology, BioPharmaceuticals R&D, Gothenburg, Sweden; 4 Translational Genomics, Discovery Biology, Discovery Sciences, AstraZeneca, BioPharmaceuticals R&D, Gothenburg, Sweden; 5 Data Sciences and Quantitative Biology, Discovery Sciences, R&D, AstraZeneca, Gothenburg, Sweden; 6 Clinical Development, Research and Early Development, Respiratory & Immunology, BioPharmaceuticals R&D, AstraZeneca, Gothenburg, Sweden

**Keywords:** COPD, ACE2, COVID-19, SARS-CoV-2, Infection, Inflammation

## Abstract

**Background:**

Chronic obstructive pulmonary disease (COPD) patients are at increased risk of poor outcome from Coronavirus disease (COVID-19). Early data suggest elevated Severe Acute Respiratory Syndrome Coronavirus 2 (SARS-CoV-2) receptor angiotensin converting enzyme 2 (ACE2) expression, but relationships to disease phenotype and downstream regulators of inflammation in the Renin-Angiotensin system (RAS) are unknown. We aimed to determine the relationship between RAS gene expression relevant to SARS-CoV-2 infection in the lung with disease characteristics in COPD, and the regulation of newly identified SARS-CoV-2 receptors and spike-cleaving proteases, important for SARS-CoV-2 infection.

**Methods:**

We quantified gene expression using RNA sequencing of epithelial brushings and bronchial biopsies from 31 COPD and 37 control subjects.

**Results:**

ACE2 gene expression (log2-fold change (FC)) was increased in COPD compared to ex-smoking (HV-ES) controls in epithelial brushings (0.25, p = 0.042) and bronchial biopsies (0.23, p = 0.050), and correlated with worse lung function (r = − 0.28, p = 0.0090). ACE2 was further increased in frequent exacerbators compared to infrequent exacerbators (0.51, p = 0.00045) and associated with use of ACE inhibitors (ACEi) (0.50, p = 0.0034), having cardiovascular disease (0.23, p = 0.048) or hypertension (0.34, p = 0.0089), and inhaled corticosteroid use in COPD subjects in bronchial biopsies (0.33, p = 0.049). Angiotensin II receptor type (AGTR)1 and 2 expression was decreased in COPD bronchial biopsies compared to HV-ES controls with log2FC of –0.26 (p = 0.033) and − 0.40, (p = 0.0010), respectively. However, the AGTR1:2 ratio was increased in COPD subjects compared with HV-ES controls, log2FC of 0.57 (p = 0.0051). Basigin, a newly identified potential SARS-CoV-2 receptor was also upregulated in both brushes, log2FC of 0.17 (p = 0.0040), and bronchial biopsies, (log2FC of 0.18 (p = 0.017), in COPD vs HV-ES.

Transmembrane protease, serine (TMPRSS)2 was not differentially regulated between control and COPD. However, various other spike-cleaving proteases were, including TMPRSS4 and Cathepsin B, in both epithelial brushes (log2FC of 0.25 (p = 0.0012) and log2FC of 0.56 (p = 5.49E−06), respectively) and bronchial biopsies (log2FC of 0.49 (p = 0.00021) and log2FC of 0.246 (p = 0.028), respectively).

**Conclusion:**

This study identifies key differences in expression of genes related to susceptibility and aetiology of COVID-19 within the COPD lung. Further studies to understand the impact on clinical course of disease are now required.

**Supplementary Information:**

The online version contains supplementary material available at 10.1186/s12931-021-01755-3.

## Background

Coronavirus disease (COVID-19) is a heterogeneous disease with variable clinical outcomes ranging from asymptomatic disease to severe pneumonia and multi-organ failure [1–4]. Whilst the overall mortality risk is less than 1% of cases, this varies considerably with age and comorbidities associated with worse outcome [1, 5, 6]. Both cardiovascular and respiratory disease have been identified as individual risk factors for hospitalisation and death [5, 7, 8]. However, currently, there is no conclusive evidence of an increased incidence of COVID-19 in patients with chronic obstructive pulmonary disease (COPD) [9, 10]. A study reporting outcomes from the first disease wave in China has, however, identified that COPD carried an increased risk of intensive care admission, ventilation and death, which was significant even after adjustments for age and smoking were made [1]. Furthermore, two systematic reviews have now confirmed COPD to be significantly associated with severe COVID-19 outcomes [11, 12]. The elevated risk of poor outcome is likely to be greatly underestimated due to the high prevalence of undiagnosed COPD internationally [13, 14].

The factors driving susceptibility to severe acute respiratory syndrome coronavirus 2 (SARS-CoV-2) infection and the development of severe COVID-19 are complex and at present poorly understood. Smokers and COPD patients demonstrate increased levels of the SARS-CoV-2 spike protein cellular receptor, Angiotensin I Converting Enzyme 2 (ACE2) RNA, in the respiratory epithelium [15]. How these receptor levels relate to disease severity or endotype however remains uncertain. Other co-receptors including neuropilin-1 (NRP1) have also recently been shown to bind furin-cleaved substrates and increase SARS-CoV-2 infectivity [16]. Cluster of differentiation (CD)147 (basigin) has similarly been shown to bind SARS-CoV-2 spike protein, but a role in SARS-CoV-2 infection has not been demonstrated [17]. The regulation of these receptors in COPD and potential role in susceptibility to worse COVID-19 outcomes, is not yet understood.

Prior to ACE2 binding the virus relies on priming of the spike protein by the transmembrane proteases, serine 2 (TMPRSS2) and 4 (TMPRSS4) [18]. Whilst it is understood that protease activity is deranged in the COPD airway [19, 20], the expression of these specific proteases in the respiratory epithelium in COPD is not known. Other proteases have also recently been suggested to play a role in SARS-CoV-2 infection including Furin, Cathepsins B and Cathepsin L [21].

Beyond susceptibility to infection there is an emerging narrative of excessive pulmonary inflammation in severe COVID-19. COPD has long been known to be associated with abnormal inflammatory responses to viral infection which manifest as acute exacerbations [22, 23], the major driver of morbidity and healthcare costs [24]. This deleterious inflammatory response may be influenced by both innate [25, 26] and adaptive immune dysregulation [27]. In the context of SARS-CoV-2 infection, the renin-angiotensin system (RAS) has been identified as a potential driver to pulmonary and systemic immune responses. The physiological balance between pro-inflammatory, vasoconstrictive and pro-fibrotic effects of angiotensinogen through the receptor angiotensin II receptor type (AGTR)1 is maintained in health by an equipoise with AGTR2 and by Mas receptor engagement by Angiotensin 1–7 [28–30]. This balance may be lost in COPD and, along with altered ACE2 and protease expression, lead to not only increased SARS-CoV-2 susceptibility but an aberrant and impactful inflammatory response.

We investigated these mechanisms by studying key transcriptomic profiles in lung tissue and airway epithelium of deeply phenotyped COPD patients and COPD endotypes as well as ex-smoking and non-smoking controls, to provide novel insights into COVID-19 susceptibility in COPD and potential routes to developing new therapeutic strategies.

## Materials and methods

### Subjects

Subjects recruited included control ex-smokers (HV-ES) (n = 20) and patients with stable, mild or moderate COPD as defined by GOLD guidelines (n = 31), all of which had stopped smoking at least 6 months prior and had at least a 10-pack year history. Control never-smokers (HV-NS) were also recruited for comparison (n = 17). Post-bronchodilator spirometry was used to assess airflow obstruction with a forced expiratory volume in 1 s (FEV1)/ forced vital capacity (FVC) ratio of < 0.7 and an FEV1 of ≥ 50% predicted value required for enrolment as COPD subjects. Exclusion criteria included a history of other pulmonary disease, α-1-antitrypsin deficiency, long-term antibiotics/steroids, or an exacerbation within the month prior to recruitment.

For additional sub-group analysis, COPD subjects were split by endotype into different groups dependent on exacerbation frequency, COPD subjects were classified as either infrequent exacerbators (P-IE) (≤ 1 exacerbation in the preceding 12 months before enrolment) (n = 17) or frequent exacerbations (P-FE) (≥ 2 exacerbations in the preceding 12 months before enrolment) (n = 14). Sub-group analysis was also performed on patients dependent on having a history of blood eosinophilia or not. A history of blood eosinophilia was defined as having had a prior recorded blood eosinophil count of ≥ 0.3 × 10^9^/L or not (n = 23 eosinophilic and n = 7 non-eosinophilic, respectively).

Bronchoscopy sampling was performed on an outpatient basis and was approved by and performed in accordance with National Research Ethics Service South Central ethical standards – Hampshire A and Oxford C Committees (LREC no: 15/SC/0528). Patients underwent fibre optic bronchoscopy with two lobes sampled per subject. Epithelial brushings and bronchial pinch biopsies were taken from each lobe for RNA extraction and sequencing. Care was taken not to contaminate the brushes with excess blood.

### RNA sequencing

Total RNA was extracted from epithelial brushing and bronchial biopsy samples (Additional file 1: Table S1) using the AllPrep DNA/RNA/miRNA Universal Kit (Qiagen). The quantity and quality of RNA samples were determined using the standard RNA analyzer kit on a 96-channel Fragment analyzer (Agilent Technologies). Extracted samples with a yield concentration > 25 ng/µl total RNA, and a DV_200_ value (percentage of RNA fragments > 200nucleotides) >  = 30% were deemed to be of sufficient quantity and quality for TotalRNA-seq analysis. Samples were diluted to 25 ng/µl using a Tecan Fluent liquid handling automation system (Tecan). Library preparation was done in four separate runs, one 96 well plate per run. The Kapa RNA HyperPrep Kit with RiboErase (HMR) was used for reverse transcription, generation of double stranded cDNA and subsequent library preparation and indexing to facilitate multiplexing (Roche), all of which was performed through automation on a Tecan fluent. The libraries were quantified with the 96-channel Fragment Analyzer using the standard sensitivity next generation sequencing (NGS) kit (Agilent Technologies). Samples from each preparation plate were pooled and the final pools (4 in total) were quantified using a Qubit instrument for concentration determination with the DNA High Sensitivity kit (ThermoFisher Scientific). Fragment size was determined using the Fragment Analyzer, standard sensitivity NGS kit (Agilent Technologies). Three of four library pools were further diluted to 1 nM and sequenced on a NovaSeq 6000 (Illumina) using NovaSeq 6000 S4 Reagent Kit, 2 × 76 cycles. The remaining library pool was diluted to 1.9 nM and sequenced on NovaSeq 6000 (Illumina) using 2 NovaSeq 6000 SP S1 Reagent Kits, 2 × 51 cyclers. Average reads per sample were 52.6 million.

### Bioinformatics and statistical analysis

Fastq files from 245 paired-end sequencing libraries generated from 120 epithelial brushings and 125 bronchial biopsies were collected and read quality for all libraries was accessed using FastQC (v0.11.7) [31], Qualimap (v2.2.2c) [32] and samtools stats (v1.9) [33]. Quality control (QC) metrics for Qualimap were based on a STAR (v2.7.2b) [34] alignment against the human genome (GRCh38, Ensembl v99). Next, QC metrics were summarized using MultiQC (v1.7) [35]. Two libraries were excluded; one due to a low mapping rate (57% vs [79%–97%]) and another due to low sequencing throughput (210 k reads vs [20 M–86 M]), leaving 118 epithelial brushings and 125 bronchial biopsies for further analysis. Sequencing adapters were then trimmed from the remaining libraries using NGmerge (v0.3) [36]. A human transcriptome index consisting of cDNA and ncRNA entries from Ensembl (v99) was generated and reads were mapped to the index using Salmon (v1.1.0) [37]. The bioinformatics workflow was organized using Nextflow workflow management system (v19.07) [38] and Bioconda software management tool [39].

Differential gene expression were assessed with DESeq2 (v 1.26.0), using “normal” [40] for fold change shrinkage, all in R (v 3.6.1) [41]. Estimated counts was used as input for DESeq2 with lowly expressed genes excluded (requiring at least 10 counts in at least 20 samples, n = 27,229). In the models used to assess differential expression between subject groups, effects from gender and a technical batch-effect (library prep plate) were taken into account. To estimate the effect of a medication or comorbidity, disease state was also included in the model where possible, i.e. where medication or comorbidity in question had “Yes” and “No” calls for subjects in both the control group and COPD group.

For visualization, gene expression were plotted as variance stabilized transformation (vst) [42] on batch-corrected counts using the ComBat_seq function in the sva package (v 3.36.0) [43] in R (v 4.0.0) [41], the model for batch correction used the sample type, patient group and gender as co-variates. Plots were generated in R (v 4.0.2) using ggplot2 (v 3.3.2) and ggbeeswarm (v0.6.0) and ggpubr (v 0.4.0). The ratio of AGTR1:2 expression is estimated as the difference of the vst of AGTR1 and AGTR2, using the property of vst values approximating the logarithm of a gene’s expression, p values were reported as uncorrected. The reported fold changes of AGTR1:2 ratios are the differences of the median AGTR1:2 ratios of the respective groups. Linear regression was used to test association between gene expression (vst) and continuous variables.

Demographics data were analysed by conventional statistical packages (SPSS v27; Prism Graphpad v9.0). Comparisons between categorical variables were carried out by X-square (if count > 5) or Fisher’s exact test (if count = or < 5). Single comparisons between numerical variables were carried out by Kruskal–Wallis ANOVA analysis of the medians. For multiple comparisons, the Kruskal–Wallis test was followed by Dunn’s post-hoc correction.

## Results

### Patient demographics

ACE2 is the predominant receptor used by SARS-CoV-2 to bind and infect host cells. We therefore looked to see if expression of ACE2 and related genes was different between COPD and control subjects, and correlated ACE2 expression with physiological measures of lung function, exacerbation frequency, cardiovascular disease and use of inhaled corticosteroids (ICS) and other medications. Table 1 shows the demographic data for COPD vs HV-ES controls, with the HV-NS and COPD frequent exacerbator and infrequent exacerbator subgroups given in Table S2. No differences between COPD and HV-ES controls were seen in confirmed hypertension, use of angiotensin II receptor blockers (ARBs) or use of angiotensin converting enzyme inhibitors (ACEi). Importantly, no significant differences between COPD and HV-ES controls were seen in age (p = 1.0), gender (p = 0.060) or pack year history (p = 0.81). However, significant differences were seen between COPD and HV-ES controls in FEV1%, FEV1/FVC, ICS use, and confirmed cardiovascular disease.

**Table 1:** Demographics of healthy volunteer ex-smoker controls compared with COPD subjects

	HV-ES controls	COPD	P value
Number of patients (total = 51)	20	31	
M/F	11/9	25/6	0.06
Age	67.5, IQR = 6.75	70, IQR = 9.5	1.0
FEV1%	100.5, IQR = 11.75	73, IQR = 21	** < 0.0001**
FEV1/FVC ratio	77.5, IQR = 4.5	58, IQR = 13.5	** < 0.0001**
Pack-years of smoking	25, IQR = 18.62	44, IQR = 37.5	0.81
BMI, kg/m2	27.69, IQR = 3.61	28.48, IQR = 5.97	1.0
Inhaled corticosteroid use, n (19/68)	0	19	** < 0.0001**
ACEi use, n (7/68)	3	4	1.0
ARB use, n (6/68)	2	4	1.0
Hypertension, n (16/68)	6	10	1.0
Cardiovascular disease, n (7/68)	0	7	**0.03**
Diabetes, n (5/68)	1	4	0.63

*ARB* Angiotensin receptor blockers, *ACEi* Angiotensin-converting enzyme inhibitor, *CVD* cardiovascular disease, *BMI* body mass index, *COPD* chronic obstructive pulmonary disease, *FEV 1* forced expiratory volume in one second, *FVC* forced vital capacity. *HV-ES* health volunteer ex-smoker who had stopped smoking for at least 6 months

Data are presented as median and IQR (interquartile range) unless otherwise indicated

### ACE2 expression was increased in COPD and inversely correlated with lung function

**Fig. 1:**
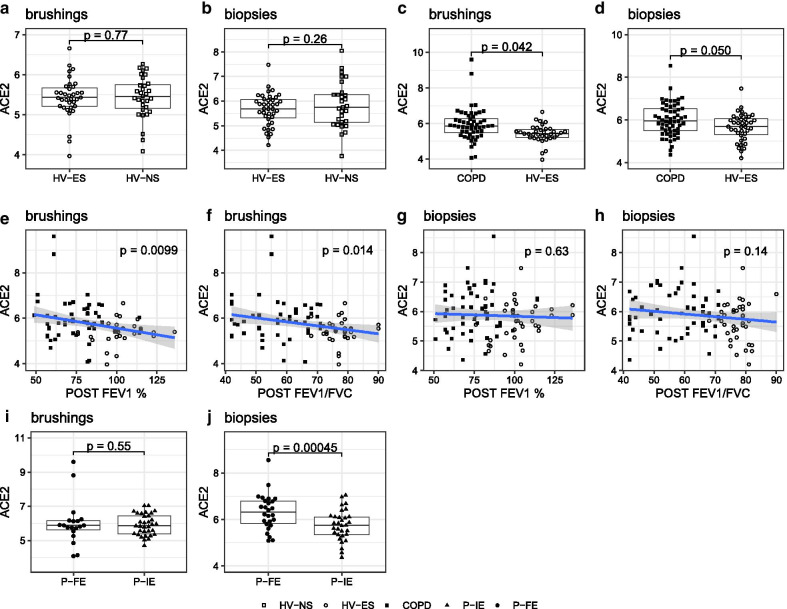
Angiotensin-converting enzyme 2 (ACE2) expression in epithelial brushings (**a, c, e, f, i**) and bronchial biopsies(**b, d, g, h, j**). Gene expression is reported in vst. Each symbol represents a single sample, generally there are two samples per subject, symbol shapes indicate the different subject groups as shown in the legend at the bottom using the definitions in the main text. Boxes illustrate the median 25th and 75th percentile, whiskers extend to the smallest or largest value that is at most 1.5 times the interquartile range from the hinge. P-values represent the results of testing for differential expression using DeSeq2. **a**–**d**, **i**–**j** Blue lines represent the best fit of the relation between ACE2 and post bronchodilator lung function measurements, the grey shaded area represents the 95% confidence interval of the fit (**e**–**h**)

We investigated if ACE2 expression was differentially regulated in COPD compared with HV-ES controls as this may increase the risk of developing COVID-19 disease [5, 44]. ACE2 expression was not differentially regulated between HV-ES controls and HV-NS in epithelial brushes and bronchial biopsies with a log2-fold change (FC) of − 0.024 (p = 0.77) and − 0.14 (p = 0.26), respectively (Fig. 1a, b). However, there was a significant increase in ACE2 expression between COPD subjects compared with HV-ES controls in both epithelial brushes and bronchial biopsies with a log2FC of 0.25 (p = 0.042) and 0.23 (p = 0.050), respectively (Fig. 1c, d).

We next looked to see if ACE2 expression correlated with physiological measures of lung function and found that increased ACE2 expression inversely correlated with FEV1% (− 0.28, p = 0.0099) (Fig. 1e) and FEV/FVC (− 0.26, p = 0.014) in epithelial brushes (Fig. 1f). However, there was no correlation between ACE2 expression and FEV1% (0.049, p = 0.63) or FEV/FVC (− 0.15, p = 0.14) in bronchial biopsies (Fig. 1g, h).

We subsequently looked to see whether ACE2 expression was different dependent on frequency of COPD exacerbations. ACE2 expression was increased in bronchial biopsies of P-FE compared with P-IE with a log2FC of 0.51 (p = 0.00045) (Fig. 1j). However, ACE2 expression was not different in epithelial brushes between P-FE and P-IE, log2FC of − 0.033 (p = 0.55) (Fig. 1i).

**Fig. 2:**
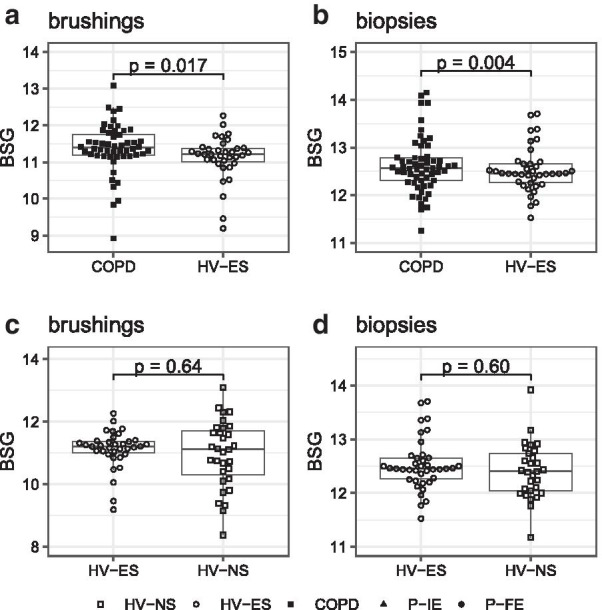
Cluster of differentiation (CD)147 (basigin) expression in epithelial brushes (**a, c**) and bronchial biopsies (**b, d**) in COPD vs HV-ES (**a, b**) and HV-ES vs HV-NS (**c, d**). Gene expression is reported in vst, which corrects for sequencing depth and applies a variance stabilizing transformation [42]. The interpretation of graphical elements is the same as in Fig. 1.  P-values represent the results of testing for differential expression using DeSeq2

We then looked at other potential SARS-CoV-2 receptors including basigin and neuropilin-1. Basigin was expressed in both brushes and bronchial biopsies and was upregulated in COPD vs HV-ES controls, log2FC of 0.17 (p = 0.0040) and log2FC of 0.18 (p = 0.017), respectively (Fig. 2a, b). However, no difference was seen in basigin expression between HV-ES vs HV-NS controls, in either epithelial brushes or bronchial biopsies, log2FC of 0.020 p = 0.64) and log2FC of 0.60 (p = 0.021) (Fig. 2c, d), respectively. Neuropilin-1 was expressed in both epithelial brushes and bronchial biopsies, but no differences were seen between controls and COPD (Additional file 1: Figure S1).

### ACE2 expression was not related to BMI, age, or gender but was increased in subjects using ACEi, subjects with cardiovascular disease and COPD subjects who use ICS

BMI and age have been found to correlate with COVID-19 disease outcomes and male gender predisposes to severe COVID-19. Furthermore, hypertension and cardiovascular disease have been shown to predispose to worse COVID-19 outcomes with the impact of ACEi and ARB still to be fully delineated. We therefore looked to see if these variables associated with ACE2 expression in the lung (Table 1). ACE2 expression did not correlate with BMI or age and was not different dependent on gender in either epithelial brushes or bronchial biopsies (data not shown). However, use of ACEi did associate with increased ACE2 expression in bronchial biopsies but not epithelial brushes with log2FC of 0.50 (p = 0.0034) and 0.026 (p = 0.82), respectively. Increased ACE2 expression did not associate with use of ARBs either in bronchial biopsies or epithelial brushes, log2FC of 0.12 (p = 0.50) and − 0.084 (p = 0.46), respectively.

ACE2 expression was increased in bronchial biopsies of subjects with cardiovascular disease or hypertension with log2FC of 0.23 (p = 0.048) and 0.34 (p = 0.0089), respectively. However, this was not seen in epithelial brushes, log2FC of − 0.011 (p = 0.85) and − 0.025 (p = 0.74), respectively. ICS use has previously been found to decrease ACE2 expression in cells from sputum in asthma [45]. In COPD subjects, use of ICS was associated with increased ACE2 expression in bronchial biopsies but not in epithelial brushes with log2FC of 0.33 (p = 0.049) and − 0.013 (p = 0.90), respectively.

### The AGTR1:AGTR2 ratio is increased in COPD

The balance of AGTR1 and AGTR2 is thought to be important for controlling the pro and anti-inflammatory responses to angiotensin signalling [28–30]. We therefore looked at the expression of related genes AGTR1 and AGTR2 and the ratio of these within the lung.

**Fig. 3:**
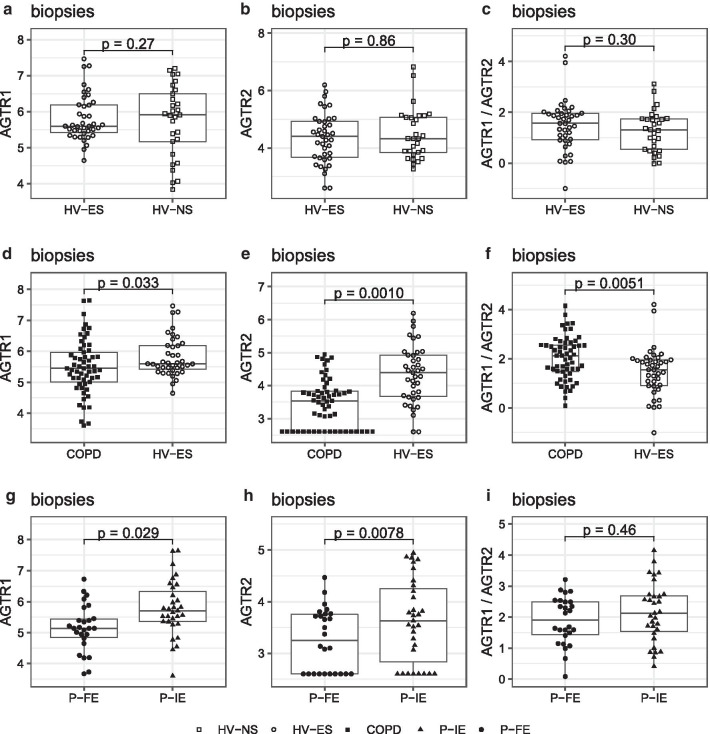
AGTR1 (**a, d, g**) and AGTR2 (**b, e, h**) expression in bronchial biopsies and the AGTR1/AGTR2 ratio (**c, f, i**), compared between HV-ES and HV-NS (**a, b, c**), COPD and HV-ES (**d, e, f**) and P-FE and P-IE (**g, h, i**). Gene expression is reported in vst, which corrects for sequencing depth and applies a variance stabilizing transformation [42]. The interpretation of graphical elements is the same as in Fig. 1. P-values represent the results of testing for differential expression using DeSeq2. **c**, **f**, **i** AGTR1/AGTR2 ratios are approximated by the difference between vst AGTR1 and AGTR2 expression

The expression of AGTR1 and AGTR2 or the AGTR1:2 ratio were not different in bronchial biopsies between HV-ES controls compared with HV-NS with log2FC of 0.12 (p = 0.27), 0.14 (p = 0.86) and 0.25 (p = 0.30), respectively (Fig. 3a-c, respectively). Neither AGTR1 nor AGTR2 were detected in the majority of brushings. Therefore, no meaningful comparison of expression was possible.

However, both AGTR1 and AGTR2 were expressed in bronchial biopsies and expression was decreased in COPD subjects compared with HV-ES controls with log2FC of − 0.26 (p = 0.033) and − 0.40 (p = 0.0010), respectively (Fig. 3d, e, respectively). The AGTR1:2 ratio was increased in COPD subjects compared with HV-ES controls with log2FC of 0.57 (p = 0.0051, Fig. 3f). Furthermore, a decrease in AGTR1 and AGTR2 expression was seen in bronchial biopsies from P-FE compared with P-IE with log2FC of − 0.37 (p = 0.029) and − 0.59 (p = 0.0078), respectively. However, a difference in the AGRT1:AGTR2 ratio was not detected with log2FC of − 0.22 (p = 0.46), (Fig. 3g–i).

**Fig. 4:**
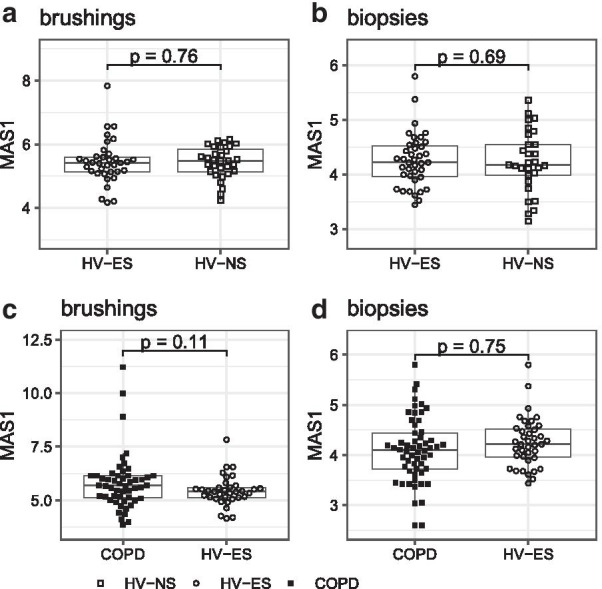
MAS1 proto-oncogene (MAS1) expression in epithelial brushes (**a, c**) and bronchial biopsies (**b** and **d**), comparing HV-ES and HV-NS (**a, b**) and COPD and HV-ES (**c, d**). Gene expression is reported in vst. The interpretation of graphical elements is the same as in Fig. 1. P-values represent the results of testing for differential expression using DeSeq2

MAS1 was also expressed within both epithelial brushes and bronchial biopsies but was not altered in expression between HV-ES controls and HV-NS with log2FC of − 0.027 (p = 0.76) and 0.038 (p = 0.69), respectively (Fig. 4a, b). Expression of MAS1 was also not different in epithelial brushes and bronchial biopsies from COPD and HV-ES controls with log2FC of 0.32 (p = 0.11) and 0.0035 (p = 0.75), respectively (Fig. 4c, d).

### Spike-cleaving protease expression in the COPD lung

**Fig. 5:**
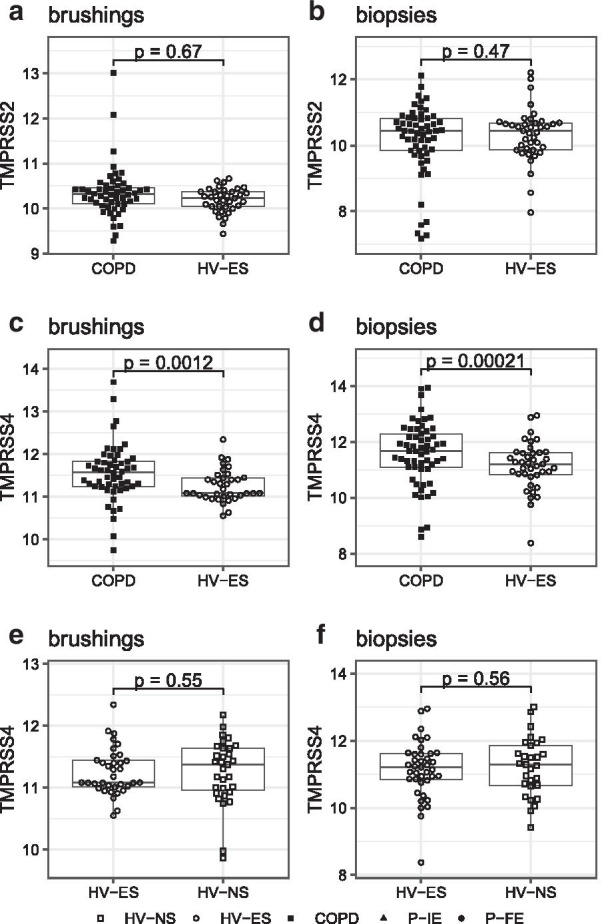
Transmembrane protease, serine 2 (TMPRSS2) (**a, b**) and 4 (TMPRSS4) (**c-f**) expression in bronchial biopsies (**b, d, f**) and epithelial brushings (**a, c, e**). Gene expression is reported in vst. The interpretation of graphical elements is the same as in Fig. 1. P-values represent the results of testing for differential expression using DeSeq2

TMPRSS2 and TMPRSS4 have been demonstrated to be important for cleavage of SARS-CoV-2 to allow entry and infection of host cells. We therefore investigated if these and other proteases were differentially regulated between health and COPD. TMPRSS2 was expressed in both epithelial brushes and bronchial biopsies but was not differentially regulated in COPD compared with HV-ES controls with log2FC of 0.023 (p = 0.67) and 0.069 (p = 0.47), respectively (Fig. 5a, b). However, TMPRSS4 was upregulated in COPD as compared with HV-ES in both epithelial brushes (log2FC of 0.25, p = 0.0012) and bronchial biopsies (log2FC of 0.49, p = 0.00021) (Fig. 5c, d), but not between HV-ES and HV-NS in either epithelial brushes (log2FC of − 0.051, p = 0.55) or bronchial biopsies log2FC of − 0.13 (p = 0.56) (Fig. 5e, f).

Other spike processing proteases were also upregulated in COPD compared with HV-ES in epithelial brushes including Cathepsin B (log2FC of 0.56, p = 5.49E−06) (Additional file 1: Figure S2A), Cathepsin L (log2FC of 0.32, p = 0.011) (Additional file 1: Figure S3A), and Furin (log2FC of 0.32, p = 0.005) (Figure S4A). However, no differences were seen between HV-ES vs HV-NS, log2FC of − 0.17 (p = 0.12) (Additional file 1: Figure S2C), log2FC of − 0.135 (p = 0.19) (Additional file 1: Figure S3C) and log2FC of 0.077 (p = 0.51) (Additional file 1: Figure S4C), respectively. Cathepsin B was also upregulated in COPD vs HV-ES in bronchial biopsies (log2FC of 0.246 p = 0.028) (Additional file 1: Figure S2B), but not in HV-ES vs HV-NS, log2FC of − 0.12 (p = 0.33) (Additional file 1: Figure S2D). However, Cathepsin L and Furin were not differentially expressed between bronchial biopsies in controls vs COPD (Additional file 1: Figure S3D and S4D).

### SARS-CoV-2 related gene expression in eosinophilic COPD

SARS-CoV-2 receptors were not differentially regulated between eosinophilic and non-eosinophil subjects with COPD. No difference was seen in expression of ACE2, neuropilin-1 or basigin or the ACE2 related receptors AGTR1, AGTR2 or MAS1 in epithelial brushes. Similarly, there were no expression differences between spike-cleaving proteases TMPRSS2, TMPRSS4, Furin, Cathepsins B or Cathepsin L in epithelial brushes (data not shown).

## Discussion

The current COVID-19 pandemic is continuing to affect the lives of individuals, health services and economies globally [1–3, 46, 47]. This study highlights that ACE2, the functional receptor for SARS-CoV-2, expression is elevated in COPD patients, as previously described [15, 48], However, we extend these previous observations to show further upregulation of this viral receptor in frequent exacerbators and those with worse lung function, as well as in COPD patients using ICS. Whilst these signals were apparent in COPD, this signal was not related to BMI or gender. We further show an upregulation of proteases relevant to SARS-CoV-2 viral fusion and entry in COPD and the newly identified basigin receptor [18, 21].

Downstream of receptor binding, the RAS pathway has been implicated in the generation of inflammatory responses to infection. The anti-inflammatory AGTR2 and pro-inflammatory, pro-fibrotic AGTR1 gated pathways are deranged in COPD with the ratio of expression favouring the inflammatory profile seen in COVID-19 pneumonia [28–30, 49]. These findings offer important insights into mechanisms of susceptibility and suggest that frequent exacerbators and those with more severe airflow obstruction are at particular risk.

The biology of SARS-CoV-2 infection is rapidly being elicited and the role of the key binding site of the main cellular receptor ACE2 is now being elucidated beyond its function in the RAS. ACE2 plays a key role in the control of vascular tone, blood pressure, tissue inflammation and repair, through the conversion of Angiotensin II to Angiotensin 1–7 [30, 50, 51]. Following SARS-CoV-2 infection, ACE2 binds to the virus via the spike protein and is internalised and so ACE2 activity may be key in affected cells [52]. ACE2 is expressed in many organ systems, particularly in lung type 2 alveolar cells [53, 54]. We demonstrate up-regulation of ACE2 and the newly identified receptor basigin in bronchial biopsies and epithelial brushes in COPD vs health controls, but no difference was seen in neuropilin-1 expression. Other potential receptors have recently been identified including LDLRAD3, TMEM30A, CLEC4G and AXL [55, 56]. Further work, delineating their role in SARS-CoV-2 infection and regulation in COPD could help further understand the susceptibility of patients with COPD to severe infection.

In our study proteases identified as important in activating the binding capacity of the spike protein were upregulated in COPD including TMPRSS4, cathepsins B and cathepsin L, but not TMPRSS2 or Furin [18, 21, 57]. Further work to try and understand the comparative role of these proteases in the COPD lung and COVID-19 pathogenesis now needs to be undertaken [19, 20].

Viral infection alone is not the only aspect of the development of severe COVID-19 as the majority of infected cases are asymptomatic or mild [2, 58]. Whilst increased infection of the lower airway may be an important factor, it is the development of excessive inflammation which is key and targets of current and potential future COVID-19 therapeutics [59–64]. This involves cytokine production [65], influx of immature monocytes [66], and T cell activation [67]. An emerging understanding of the role of the RAS in inflammatory control is also developing [30, 50, 51]. Here the engagement of AGTR2 by Angiotensin II is opposed by the activation of AGTR1 by the same ligand [68]. Many of the known consequences of AGTR1 signalling are seen in the pathology of severe COVID-19. Monocyte recruitment and activation through nuclear factor-κB and monocyte chemoattractant protein-1 signalling is seen in models of acute nephritis [69]. The origins of the cytokine storm described in more severe viral disease are multifactorial with the immature monocyte population, activated alveolar macrophages and inflammatory T cells all implicated [70]. Here the imbalance of AGTR1:2 signalling compounded by the downregulated effects of ACE2, via Angiotensin 1–7 controlled Mas receptor signalling, may be key in the development of an unopposed pro-inflammatory state seen in the pandemic and possibly acute respiratory distress syndrome (ARDS) [71].

COPD patients who suffer from frequent exacerbations express more ACE2 and may therefore experience greater risk of lung infection or more symptoms as a result of SARS-CoV-2 infection. This was not due to a past history of smoking as these effects were controlled for in the matched population. Interestingly COPD patients manifest a number of mechanisms related to susceptibility to infection and its associated inflammation beyond SARS-CoV-2. Dysregulation of other binding receptors [72, 73], impaired mucosal antibody mediated immunity [74], microbial dysbiosis [22, 25, 75, 76] and abnormal control of T cell responses [27, 77]. Similar findings of increased ACE2 expression in the sputum of asthmatics have also been reported [45, 78] and it would appear that susceptibility to the impact of respiratory viruses is therefore a key trait of vulnerable patients with airways disease. Despite the novel biology of SARS-CoV-2, it would appear this trait runs true in this study cohort.

Blood eosinophilic COPD has been demonstrated to be a stable phenotype which predicts response to corticosteroid treatment, with eosinophilic patients exhibiting the greatest response [25]. However, the role of eosinophilia in COPD exacerbations and susceptibility to viral infections has not been fully elucidated. In this study we did not see gene expression differences in SARS-CoV-2 receptors or spike proteases in patients with a blood eosinophilic endotype, indicating that these patients may not have a different predisposition to SARS-CoV-2 infection through these mechanisms. It would be useful to further investigate ACE2-related gene expression in other COPD phenotypes in future larger studies, using endotypes defined by the local inflammatory environment in the lung.

In this study we further demonstrate the increased ACE2 expression in bronchial biopsies from subjects with cardiovascular disease and hypertension, highlighting a potential mechanism impacting the widely reported increased susceptibility to COVID-19 in these patients [5]. The role of ACEi in ACE2 expression and COVID-19 susceptibility has been a topic of debate. In this study we found that ACE2 expression was increased in bronchial biopsies from participants using ACEi but not ARB. This may play a role in SARS-CoV-2 binding to host cells. However, our cohort was small, only a few patients had cardiovascular comorbidity and these patients were seen only in the COPD group, which could explain these results. Recent studies have demonstrated an overall reduced susceptibility to severe COVID-19 and mortality associated with use of ACEi and ARBs [79].

Conflicting studies using in vitro epithelial culture models have reported steroids to either increase or decrease ACE2 expression [80, 81]. In our study, sequencing RNA from samples purified directly from the COPD lung, we did not detect differences in ACE2 expression in epithelial brushes dependent on ICS use. However, we demonstrated an increased expression of ACE2 expression in bronchial biopsies, a potentially important clinical finding related to COPD patient susceptibility to COVID-19 which warrants further investigation. The increased likelihood of ICS use, particularly in COPD patients who have frequent exacerbations, could play a role in increasing their susceptibility to COVID-19 and could be a mechanism for the increased ACE2 expression we saw in FE vs IE. However, significant differences in ICS use were not seen in our cohort.

Our findings suggest differential expression levels between airway epithelial and bronchial biopsy samples. It is interesting to speculate what may drive these differences between compartments. Firstly, ACE2 expression was different in epithelial brushings and bronchial biopsies. Differential expression at the epithelial barrier interface would be a key driver to increased susceptibility, as seen in the data. Interestingly, however, AGTR expression differences were seen only in the bronchial biopsies and not in the epithelial rich brushes. Bronchial biopsies are complex tissue samples which include epithelial cells, submucosal tissue and vascular structures [82]. Previous work has identified AGTR2 but not AGTR1 expression in lung epithelium [49]. A key feature of the RAS and its role in the development of pulmonary angiopathy in COVID-19 is the involvement of the vasculature and its endothelium in the development of pathology [83]. Therefore, COPD patients may manifest susceptibility to infection at an epithelial level and also to inflammation and fibrotic change in the submucosa beneath this epithelium.

This study, like any description of disease reliant on sample analysis, has its limitations. Firstly, only relatively mild COPD patients were studied due to the limitations of bronchoscopic sampling in more severe disease. It is interesting to speculate whether our disease relevant findings would in fact be more marked in moderate to severe disease. Our control ex-smoker and COPD patients had stopped smoking at least 6 months prior to enrolment in this study. However, it is possible that duration of smoking cessation could impact gene expression and be important for COVID-19 susceptibility. Due to the intensive nature of sampling required, our cohort was relatively small and there was a non-significant increased number of male patients in our COPD arm compared to control. This could be relevant due to the increased risk of COVID-19 hospitalisation in male patients [5]. We thus cannot rule out that this difference in gender between groups could confound the gene expression differences seen in this study [5]. Whilst we were able to report clear disease relevant differences in gene expression related to COPD per se and sub phenotypes, we did not see clear effects of gender that have been described in asthma [45, 78]. We did demonstrate that cardiovascular disease and hypertension associated with increased ACE2 expression in bronchial biopsies of patients with COPD. A larger study, purposefully recruiting diabetics and heart disease patients may inform us as to the mechanisms underlying ACE2 expression in these patients and the relationship with COVID-19 severity [5]. We have been able to report differences in gene expression of key targets but have not explored protein expression or indeed experimentally determined consequences using infection models [77, 84–86]. Further work will be required to elicit the functional consequences of these findings and to ascertain the potential to modulate their effects with existing and novel treatments strategies.

## Conclusion

At a time when COVID-19 continues to cause widespread illness and premature mortality, novel insights into disease biology are precious, particularly if they improve our understanding of outcome in the most vulnerable populations. Our findings offer preliminary information highlighting a potential risk in COPD to SARS-CoV-2 infection outcome, which could be used as a roadmap to better understand outcome driven by other pathogens beyond the pandemic. However, it is uncertain whether the identified differences in COVID-19 related gene expression are directly related to clinical susceptibility and future longitudinal studies are indicated to understand this.

## Supplementary Information

**Additional file 1.** Additional figures and tables.

## Data Availability

The datasets generated and analysed during the current study are not publicly available in order to protect the privacy of all individuals whose data we have collected, stored, and analysed. However, data may be made available upon reasonable request by applying through the established Data Request Portal through which Researchers can request access to de-identified clinical data (https://vivli.org), after which, clinical data may be made available upon review of the patient consent forms, scientific merit of the proposal, and signature of a data sharing/collaboration agreement. This mechanism allows controlled, risk-managed accessibility of the data and at the same time safeguards patients’ confidentiality.

## Abbreviations

ARDS: Acute respiratory distress syndrome
ACE2: Angiotensin converting enzyme 2
ACEi: Angiotensin converting enzyme inhibitors
AGTR: Angiotensin II receptor type
ARBs: Angiotensin II receptor blockers
CD147: Cluster of differentiation 147 (basigin)
COPD: Chronic obstructive pulmonary disease
COVID-19: Coronavirus disease
FC: Log2-fold change
HV-ES: Control ex-smokers
HV-NS: Control never-smokers
ICS: Inhaled corticosteroids
NRP1: Neuropilin-1
NGS: Next generation sequencing
P-IE: COPD Infrequent exacerbators
P-FE: COPD frequent exacerbators
RAS: Renin-Angiotensin system
SARS-CoV-: Severe Acute Respiratory Syndrome Coronavirus 2
TMPRSS: Transmembrane protease, serine 2
TMPRSS: Transmembrane protease, serine 4
