# Supplementary material for: Dysregulation of COVID-19 related gene expression in the COPD lung
Source: Respir Res. 2021 May 29;22:164. doi: 10.1186/s12931-021-01755-3 (PMC8164067; doi:10.1186/s12931-021-01755-3)
Supplement: Supplementary file 1 — Additional file 1. Additional figures and tables. [file 12931_2021_1755_MOESM1_ESM.docx]

# Online Supplement

**MICA2 study group**

Bastian Angerman ^1^

Stephanie Ashenden ^2^

Sarah Bawden ^3^

Graham Belfield ^2^

Maria G. Belvisi ^1,4^

Aurelie Bornot ^2^

Jerome Bouquet ^5^

Hannah Burke ^3, 6^

Carolina Caceres ^5^

Raghothama Chaerkady ^7^

Doriana Cellura ^3, 6^

Chia-Chien Chiang ^8^

Kerry Day ^3, 6^

Antonio DiGiandomenico ^5^

Hanna Duàn ^1^

Ulrika Edvardsson ^9^

Damla Etal ^2^

Anna Freeman ^3, 6^

Matthew Glover ^7^

Vancheswaran Gopalakrishnan ^5^

Stephen Harden ^10^

Sonja Hess ^7^

Alex Hicks ^3, 6^

Ventzi Hristova ^7^

Michael Hühn ^1^

Fredrik Karlsson ^2^

Shameer Khader ^8^

Glenda Lassi ^1^

Alex Mackay ^1^

Chris McCrae ^1^

Christopher Morehouse ^5^

Daniel Muthas ^1^

Karl Nordström ^2^

Steven Novick ^2^

Esther Nyimbili ^3^

Kristoffer Ostridge ^1, 6^

Lisa Öberg ^1^

Adam Platt ^1^

Laura Presland ^3^

Xiaotao Qu ^8^

Nicola Rayner ^3^

Pedro Rodrigues ^3^

Bret Sellman ^5^

Gary Sims ^1^

Cosma Mirella Spalluto ^6^

Andria Staniford ^3^

Karl J. Staples ^3, 6^

Bruce Thompson ^11^

Junmin Wang ^7^

Paul Warrener ^5^

Alastair Watson ^6^

Nicholas P. Williams ^3, 6^

Tom M. A Wilkinson ^3, 6^

Wen Yu ^8^

Bairu Zhang ^2^

Tianhui Zhang ^2^

Natalie van Zuydam ^2^

^1^ Research and Early Development, Respiratory & Immunology, BioPharmaceuticals R&D, AstraZeneca, Gothenburg, Sweden

^2^ Discovery Sciences, BioPharmaceuticals R&D, AstraZeneca, Gothenburg, Sweden

^3^ NIHR Southampton Biomedical Research Centre, Southampton, UK

^4^ Respiratory pharmacology, National Heart & Lung Institute, Imperial College London, London, UK

^5^ Microbial sciences, BioPharmaceuticals R&D, AstraZeneca, Gothenburg, Sweden

^6^ Faculty of Medicine, University of Southampton, Southampton, UK

^7^ Antibody Discovery and Protein Engineering, BioPharmaceuticals R&D, AstraZeneca, Gothenburg, Sweden

^8^ Data science and artificial intelligence, BioPharmaceuticals R&D, AstraZeneca, Gothenburg, Sweden

^9^ Business Development and licensing, BioPharmaceuticals R&D, AstraZeneca, Gothenburg, Sweden

^10^ University Hospital Southampton NHS Foundation Trust, Southampton, UK

^11^ Swinburne University of Technology Melbourne, Australia

**Supplemental tables**

Table S1 Samples analysed in this study

| **Patient group**  **(n = patients)** | **Epithelial brushings (n = samples)** | | **Bronchial biopsies (n = samples)** | |
| --- | --- | --- | --- | --- |
|  | **Pre-QC** | **Post-QC** | **Pre-QC** | **Post-QC** |
| HV-NS (17) | 33 | 31 | 30 | 29 |
| HV-ES (20) | 40 | 35 | 39 | 39 |
| P-IE (17) | 34 | 33 | 31 | 31 |
| P-FE (14) | 28 | 19 | 26 | 26 |
| Total (68) | 135 | 118 | 126 | 125 |

Table S1. describes the numbers of epithelial brushings and bronchial biopsy samples from the different patient groups (HV-ES, HV-NS, P-FE & P-IE) that were generated (Pre-QC) and those of sufficient RNA quality (DV200 value >= 30%) to be processed for RNA-seq (post-QC)

Table S2. Subject demographics

|  | **Control** | | | **COPD** | | | **Control vs. COPD*** |
| --- | --- | --- | --- | --- | --- | --- | --- |
|  | HV-NS | HV-ES | P Value  (HV-NS vs HV-ES) | P-IE | P-FE | P Value  (P-IE vs P-FE) | P value |
| N of patients (TOT=68) | 17 | 20 | - | 17 | 14 | - | - |
| M/F | 10/7 | 11/9 | 1.0 | 13/4 | 12/2 | 0.66 | **0.04** |
| Age | 63, IQR=11 | 67.5, IQR=6.75 | 1.0 | 70, IQR=9 | 71, IQR=13.25 | 1.0 | 1.0 |
| FEV1% | 103, IQR= 11 | 100.5, IQR= 11.75 | 1.0 | 73, IQR= 21 | 71.5, IQR= 18 | 1.0 | **<0.0001** |
| FEV1/FVC ratio | 80, IQR= 4 | 77.5, IQR= 4.5 | 1.0 | 57, IQR= 16 | 58.5, IQR= 11.25 | 1.0 | **<0.0001** |
| Pack-years of smoking | 0.0, IQR= 1.50 | 25, IQR= 18.62 | **0.0004** | 51, IQR= 22 | 29.5, IQR= 33.94 | 1.0 | **<0.0001** |
| BMI, kg/m2 | 27.94,  IQR= 5.55 | 27.69, IQR= 3.61 | 1.0 | 27.80, IQR= 6.03 | 29.73,  IQR= 6.52 | 1.0 | 1.0 |
| Inhaled corticosteroid use, n (20/68) | 1 | 0 | 0.46 | 8 | 11 | 0.14 | **<0.0001** |
| ACEi use, n (11/68) | 4 | 3 | 0.68 | 1 | 3 | 0.30 | 0.74 |
| ARB use, n (6/68) | 0 | 2 | 0.49 | 2 | 2 | 1.0 | 0.40 |
| Hypertension, n (22/68) | 6 | 6 | 1.0 | 5 | 5 | 1.0 | 1.0 |
| Other heart disease, n (7/68) | 0 | 0 | 1.0 | 1 | 6 | **0.03** | **0.003** |
| Diabetes, n (6/68) | 1 | 1 | 1.0 | 2 | 2 | 1.0 | 0.40 |

*Definition of abbreviations: ARB= Angiotensin receptor blockers; ACEi = Angiotensin-converting enzyme inhibitor; CVD= cardiovascular disease; BMI = body mass index; COPD = chronic obstructive pulmonary disease, FEV 1 = forced expiratory volume in one second, FVC = forced vital capacity, never-smoker, HV-NS = healthy volunteer non-smokers,* *HV-ES = health volunteer ex-smoker who had stopped smoking for at least 6 months, P-IE = COPD infrequent exacerbator, P-FE = COPD frequent exacerbator.*

**Comparison for Control (all HV-NS and HV-ES combined) vs COPD (all P-IE vs P-FE combined). There is an expected difference in smoking pack year history between these two groups which is driven by the never smokers being included in control group as part of this comparison.*

*Data are presented as median and IQR (interquartile range) unless otherwise indicated.*

**Supplemental Figures**


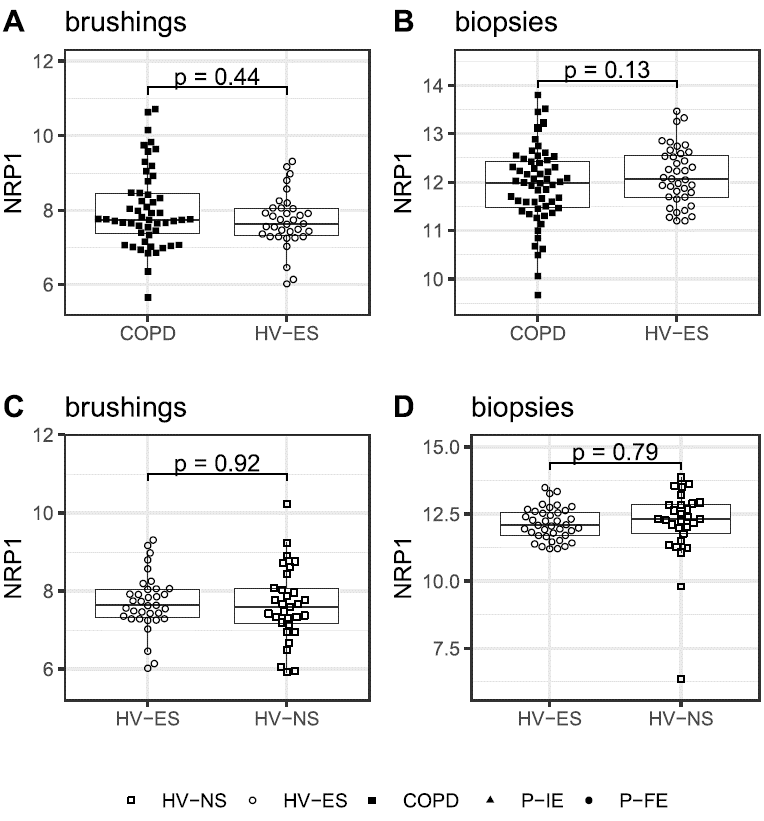


**Figure S1**

Neuropilin-1 (NRP1) expression in bronchial biopsies and brushes. Gene expression is reported in vst, which corrects for sequencing depth and applies a variance stabilizing transformation (40). The interpretation of graphical elements is the same as in Figure 1. P-values represent the results of testing for differential expression using DeSeq2.


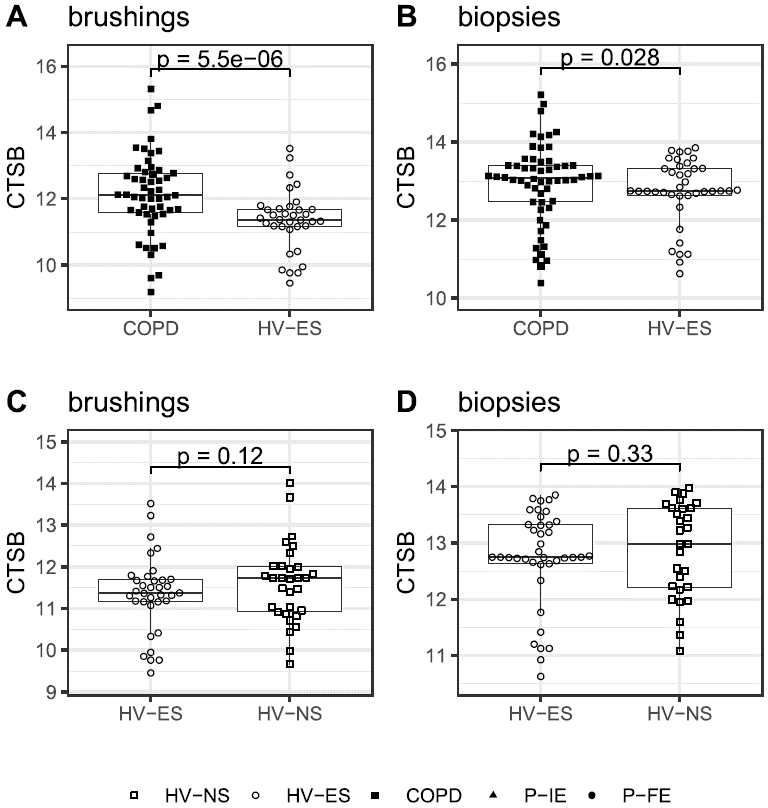


**Figure S2**

Cathepsin B (CTSB) expression in bronchial biopsies and brushes. Gene expression is reported in vst, which corrects for sequencing depth and applies a variance stabilizing transformation (40). The interpretation of graphical elements is the same as in Figure 1. P-values represent the results of testing for differential expression using DeSeq2.


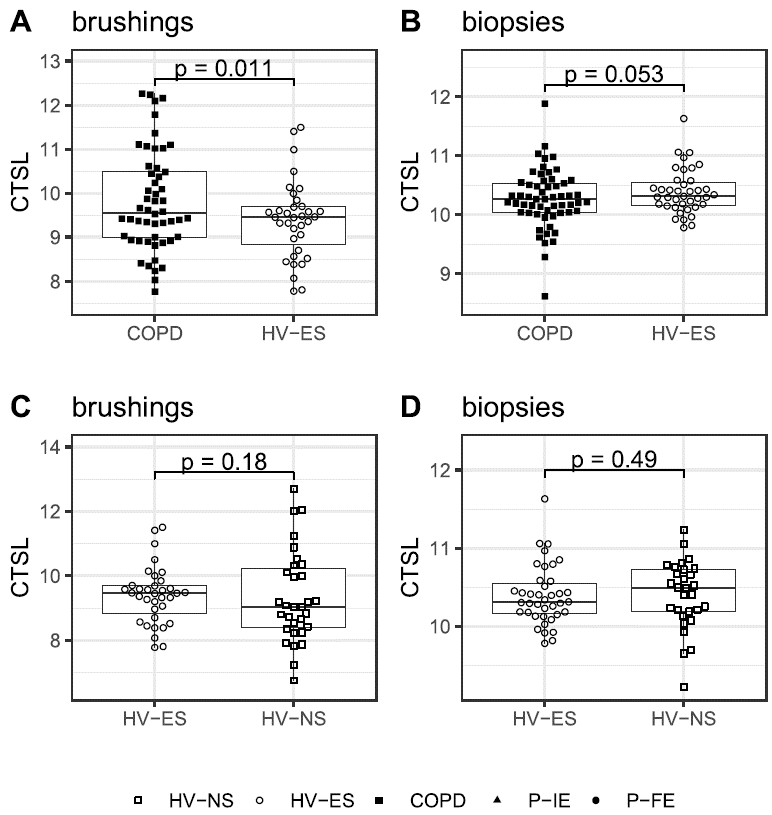


**Figure S3**

Cathepsin L (CTSL) expression in bronchial biopsies and brushes. Gene expression is reported in vst, which corrects for sequencing depth and applies a variance stabilizing transformation (40). The interpretation of graphical elements is the same as in Figure 1. P-values represent the results of testing for differential expression using DeSeq2.


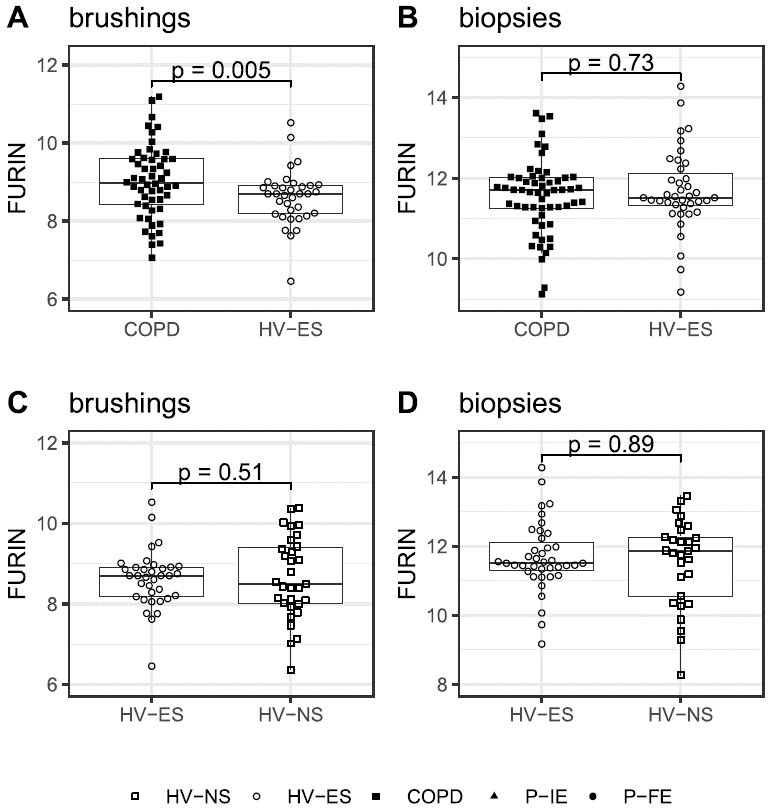


**Figure S4**

Furin expression in bronchial biopsies and brushes. Gene expression is reported in vst, which corrects for sequencing depth and applies a variance stabilizing transformation (40). The interpretation of graphical elements is the same as in Figure 1. P-values represent the results of testing for differential expression using DeSeq2.
